# GNAQ inhibits tumorigenesis via the ARHGEF25-mediated RHOA pathway in NK/T-cell lymphoma

**DOI:** 10.1080/15384047.2025.2598074

**Published:** 2025-12-09

**Authors:** Yuyang Gao, Zihe Zhang, Yue Song, Wenting Song, Hongwen Li, Lei Zhang, Zhaoming Li, Mingzhi Zhang

**Affiliations:** a Department of Oncology, The First Affiliated Hospital of Zhengzhou University, Zhengzhou, Henan, People's Republic of China; b State Key Laboratory of Esophageal Cancer Prevention & Treatment and Henan Key Laboratory for Esophageal Cancer Research, The First Affiliated Hospital of Zhengzhou University, Zhengzhou, Henan, People's Republic of China; c Department of Dermatovenereology, The Seventh Affiliated Hospital of Sun Yat-Sen University, Shenzhen, People's Republic of China

**Keywords:** Natural killer/T-cell lymphoma, GNAQ, RHOA, ARHGEF25, tumorigenesis

## Abstract

**Background:**

Natural killer/T-cell lymphoma (NKTCL) presents highly aggressive clinical behaviour, and the outcomes for relapsed and refractory patients are still poor. Our previous study identified somatic mutations in GNAQ in 8.7% of cases through whole-exome sequencing, revealing the T96S mutation in the Gαq protein.

**Materials:**

The proliferation, gemcitabine sensitivity and apoptosis of NKTCL cells were assessed by CCK-8 assays and flow cytometry. The downstream pathways of GNAQ were explored by mRNA sequencing, Western blotting and co‑immunoprecipitation. Additionally, we investigated the role of GNAQ in the activation of the RHOA pathway in NKTCL.

**Results:**

We found that GNAQ significantly inhibited the aggressive function of NKTCL, whereas the T96S mutation abolished the ability of wild-type GNAQ to trigger cell apoptosis. Further investigation revealed that GNAQ modulated NKTCL cell functions through the activation of the RHOA pathway, which is regulated by the GNAQ-ARHGEF25 complex. Clinically, high expression of RHOA was associated with improved overall survival (HR = 0.317, 95% CI: 0.126–0.800, *p* = 0.015), whereas low expression of RHOA was correlated with poorer survival outcomes. The application of an RHOA pathway inhibitor or reactivation of the RHOA pathway significantly affected the biological functions of NKTCL cells both in vitro and in vivo.

**Conclusion:**

In summary, RHOA is a critical downstream effector of GNAQ in NKTCL. GNAQ promotes RHOA activation through ARHGEF25, which in turn regulates cellular functions by modulating cell proliferation and apoptosis, thereby influencing the progression of NKTCL.

## 1 Introduction

Natural killer/T–cell lymphoma (NKTCL) is a rare lymphoma subtype of peripheral T/NK–cell lymphoma that generally exhibits highly aggressive clinical behaviour and poor prognosis, with an increased prevalence in Asia and South America.^1^^,^^2^ Although combination chemotherapy regimens that involve L–asparaginase, such as DDGP,^3^ SMILE,^4^ and *P*-GemOx^5^ and radiotherapy have been shown to improve the complete remission (CR) rate and overall survival (OS), the prognosis for patients with relapsed or refractory disease remains poor.^6^

Tumour suppressor genes play a vital role in regulating cell growth, proliferation, and differentiation by exerting negative control over these processes. Increasing evidence underscores the significant contribution of genetic factors to the pathogenesis of NKTCL.^7^^,^^8^ G–protein–coupled receptors (GPCRs) are crucial proteins in cell signalling, in which their coupling with G proteins elicits biological effects and modulates a wide range of physiological and pathological processes.^9^^,^^10^ Numerous studies have demonstrated the involvement of GPCRs, G proteins, and their downstream signalling molecules in tumour development and progression.^11^ Mutations in G protein subunits, such as GNAQ, GNAS, GNA11, and GNA12, which play central roles in cellular signal transduction, have been implicated in tumorigenesis.^12^

RHOA is a key signal transduction molecule within cells and belongs to the small G protein family of the Rho subfamily, which includes RHOA, RhoB, and RhoC.^13^ As a small G protein with GTPase activity, RHOA plays a crucial role in regulating the cytoskeletal rearrangements of microfilaments and microtubules through various signalling pathways.^14^ Additionally, RHOA mutations have been implicated in the progression of angioimmunoblastic T–cell lymphoma.^15^ However, its specific role and function in NKTCL have not yet been reported.

In our previous study, whole–exome sequencing of 127 NKTCL patients revealed that 8.7% harboured somatic mutations in GNAQ, specifically the T96S mutation in the Gαq protein,^16^ and GNAQ T96S mutations were associated with advanced tumour stage and inferior clinical outcomes in NKTCL. Functionally, the depletion of Gαq was found to increase NK cell survival, as demonstrated in conditional knockout mice. Interestingly, unlike in other solid tumours, Gαq significantly inhibited the downstream AKT and ERK pathways in NKTCL, suggesting that Gαq may exert its effects through alternative signalling pathways in this unique context.

In this study, we explored the role of GNAQ in NKTCL and investigated its downstream pathways in regulating the function of NKTCL cells. Notably, our findings revealed that GNAQ exerted its effects through the RHOA pathway, with low RHOA expression in NKTCL correlated with poor clinical prognosis. Furthermore, we demonstrated that GNAQ targeted RHOA in vitro, regulating cell proliferation and apoptosis via the ROCK1 signalling pathway. These findings highlight GNAQ and RHOA as potential tumour suppressors in NKTCL and suggest promising avenues for the development of targeted therapies for this disease.

## 2 Results

### 2.1 GNAQ influences the aggressive biological functions of NKTCL cells

**Figure 1.**
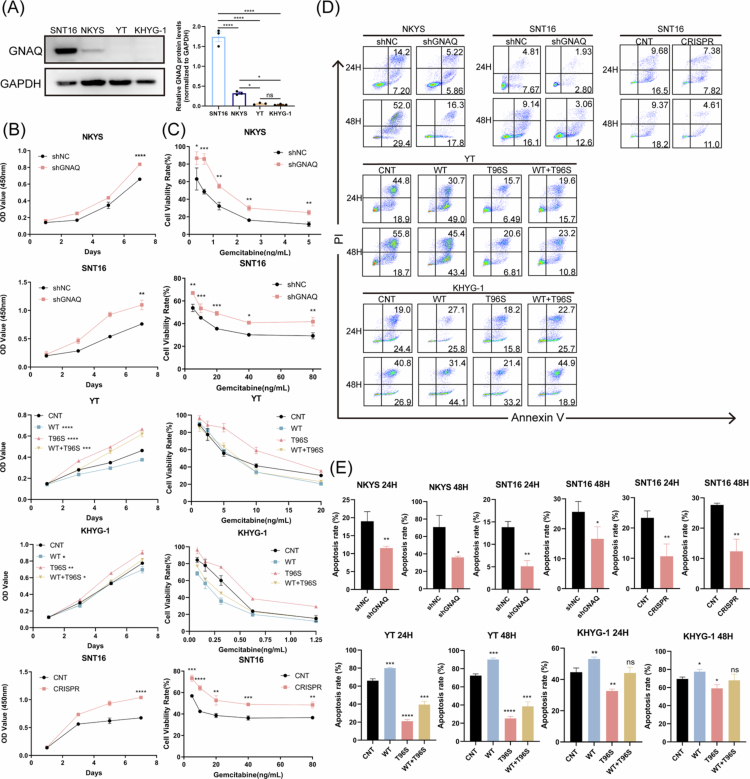
GNAQ would influence the aggressive biological functions of NKTCL cells. A. GNAQ expression was examined in 4 NKTCL cell lines by western blotting. B. Cell proliferation was detected by CCK-8. C. Gemcitabine sensitivity was detected by CCK-8. D. Starvation–induced apoptosis was detected by flow cytometry. E. Starvation–induced apoptosis was analysed. NKTCL, natural killer/T cell lymphoma; CCK-8, Cell Counting Kit-8; OD, optical density; ns, no significance; *P＜0.05; **P＜0.01; ***P＜0.001; ****P＜0.0001.

Our previous investigation revealed that GNAQ suppresses tumour growth in NKTCL, whereas the GNAQ T96S mutation may act in a dominant–negative manner to promote tumour growth. In this study, we examined the expression of endogenous GNAQ in various NKTCL cell lines (Figure 1A), noting whether the expression levels were relatively high or low. On the basis of these results, we selected NKYS and SNT16 cells to generate shGNAQ knockdown lines and YT and KHYG-1 cells to generate GNAQ wild–type (WT) and GNAQ T96S mutant lines. Additionally, we established the SNT16 cell line with a CRISPR/Cas9-mediated GNAQ c.286A > T mutation, which simultaneously expressed equal levels of wild–type and mutant GNAQ (Figure S1), as all GNAQ mutation–positive NKTCL cases are heterozygous for this mutation.

Subsequently, cell proliferation, apoptosis resistance, and gemcitabine sensitivity were assessed in vitro. As expected, cells with high GNAQ expression, including NKYS^shNC^ cells, SNT16^shNC^ cells, YT^OE–GNAQ^ cells and SNT16^OE–GNAQ^ cells, presented reduced NKTCL cell proliferation (Figure 1B), increased gemcitabine sensitivity (Figure 1C), and significant induction of starvation–induced apoptosis (Figure 1D, E). In contrast, silencing or mutating GNAQ stimulated NKTCL cell proliferation, gemcitabine resistance and apoptosis resistance in vitro.

### 2.2 GNAQ affects the RHOA pathway in NKTCL cells and is associated with the clinical prognosis of NKTCL patients

**Figure 2.**
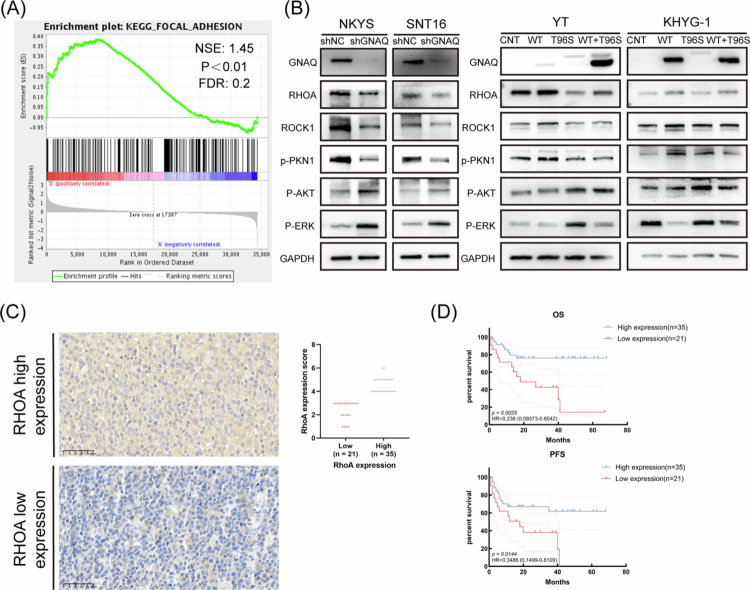
GNAQ affects the RHOA pathway in NKTCL cells and is associated with the clinical prognosis of NKTCL patients. A. GSEA analysis between NKYS^shNC^ and NKYS^shGNAQ^ cells affects the enrichment of genes in regulatory of RHOA pathway. B. Relative protein expression of RHOA pathway–related proteins was detected by western blotting. C. Typical IHC staining (40X) and RHOA expression scores of high and low RHOA expression of the NKTCL patients. D. Kaplan–Meier analysis of the correlation between the OS and PFS with the RHOA expression. NKTCL, natural killer/T cell lymphoma; GSEA, Gene set enrichment analysis; IHC, immunohistochemistry; OS, overall survival; PFS, progression–free survival; ns, no significance; *P＜0.05; **P＜0.01; ***P＜0.001; ****P＜0.0001.

The underlying mechanism of GNAQ–regulated biological function in NKTCL remains unclear. To address this issue, we performed RNA sequencing on RNA extracted from NKYS cells stably expressing either a vector control or shGNAQ. Gene set enrichment analysis (GSEA) revealed that the RHOA signalling pathway was significantly enriched in the vector control cells compared with the shGNAQ cells (NES = 1.45, FDR = 0.2) (Figure 2A). Therefore, we further investigated the expression of RHOA pathway proteins in vitro (Figure 2B, Figure S2). Collectively, the results suggest that GNAQ inhibits the activation of the downstream RHOA pathway.

We next evaluated RHOA expression in tumour tissues from 56 NKTCL patients by immunohistochemistry (Figure 2C). Patients with low RHOA expression showed poorer prognosis, including shorter overall survival (OS) and progression–free survival (PFS) (Figure 2D). RHOA expression was also significantly associated with the Prognostic Index for Natural Killer lymphoma (PINK) score (*p* = 0.042) (Table 1), suggesting a link to disease progression.

**Table 1.** Clinical features and their correlations with RhoA expression of enroled NKTCL patients.

Clinical features	N	RhoA high expression	RhoA low expression	*P* value
Total	56	35	21	
Gender				0.434
Male	45	27	18	
Female	11	8	3	
Age (years)				0.093
>60	12	5	7	
≤60	44	30	21	
B symptoms				0.835
Absent	31	19	12	
Present	25	16	9	
CA staging				0.89
I–II	30	19	11	
III–IV	26	16	10	
PINK score				0.042
0−1	33	17	16	
2−5	23	18	5	
EBV				0.89
Absent	26	16	10	
Present	30	19	11	
Serum LDH level				0.945
Normal	27	17	10	
Elevated	29	18	11	
Serum β2-MG level				0.067
Normal	40	28	12	
Elevated	16	7	9	
Ki-67 expression				0.7646
>50	38	24	14	
≤50	18	11	7	
Bone marrow invasion				0.6627
Absent	44	28	16	
Present	14	8	6	

NKTCL, natural killer/T cell lymphoma; N, number; CA, the Chinese Southwest Oncology Group and Asia Lymphoma Study Group ENKTL; PINK, Prognostic Index for Natural Killer lymphoma; LDH, lactate dehydrogenase, serum LDH was defined as >245 U/L; β2-MG, β2-microglobulin.

In univariate analysis, high RHOA expression was associated with improved PFS (HR = 0.466, 95% CI: 0.220–0.988, *p* = 0.046) and OS (HR = 0.276, 95% CI: 0.113–0.676, *p* = 0.005). After adjusting for disease stage in multivariate analysis, RHOA remained an independent favourable prognostic factor for both PFS (HR = 0.432, 95% CI: 0.202–0.925, *p* = 0.03) and OS (HR = 0.317, 95% CI: 0.126–0.800, *p* = 0.015) (Supplementary Tables S1, S2). Together, these results establish RHOA as an independent prognostic marker for improved survival in NKTCL.

### 2.3 GNAQ regulates biological functions by binding to ARHGEF25 to activate the RHOA pathway

**Figure 3.**
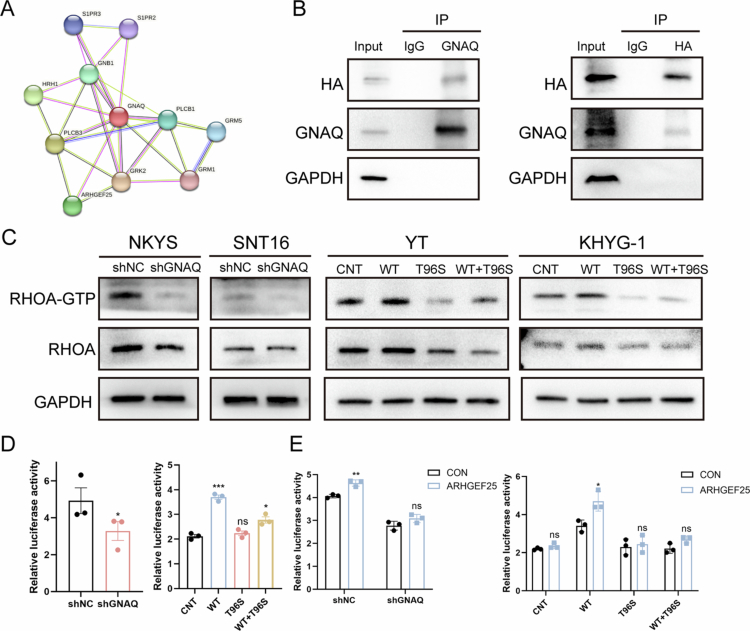
GNAQ regulates biological functions by binding to ARHGEF25 to activate the RHOA pathway. A. Network representation of STRING interactions plotted for GNAQ anti–correlated genes with lines indicating interconnectedness in terms of co–expression or interaction. B. Co–immunoprecipitation for the interaction of GNAQ and HA–tagged ARHGEF25 in SNT16 cells. C. GNAQ can influence the activation of RhoA. D. SRF luciferase assay in 293 T cells transfected with shGNAQ, WT or T96S. Relative Renilla luciferase activity was determined and normalised against the firefly luciferase activity. E. SRF luciferase assay in 293 T cells transfected with ARHGEF25 and shGNAQ, WT or T96S. Relative Renilla luciferase activity was determined and normalised against the firefly luciferase activity. *P＜0.05; **P＜0.01; ***P＜0.001; ****P＜0.0001.

The GNAQ interaction network, derived from protein‒protein interactions in STRING, highlighted key members of the potential pathway, including ARHGEF25 (Figure 3A). To evaluate the interaction between GNAQ and ARHGEF25, an HA–ARHGEF25-overexpressing SNT16 cell line was established and analysed by coimmunoprecipitation (Figure 3B). Furthermore, RHOA–GTP levels were monitored in the different cell lines with an RHOA–based assay. Notably, the results revealed that RHOA and RHOA–GTP levels were significantly elevated in GNAQ OE–WT cells, whereas silencing or mutating GNAQ led to reduced levels of these proteins (Figure 3C). Considering the crucial role of RHOA as a transcription factor that regulates the transcriptional activation of its target genes, we next sought to verify SRF–mediated transactivation in NKTCL cells. As expected, wild–type GNAQ increased the relative luciferase activity, whereas shGNAQ suppressed it (Figure 3D). Compared with the wild–type GNAQ, the mutant GNAQ clearly significantly suppressed luciferase activity, suggesting that this mutation impairs the function of GNAQ in transcriptional regulation. Moreover, compared with that in the group without wild–type GNAQ, the luciferase activity in the mutant GNAQ group was relatively lower, indicating that GNAQ retained partial function despite the presence of this novel variant. Intriguingly, the luciferase activity of wild–type GNAQ increased with increasing ARHGEF25 levels, whereas no effect was observed in the shGNAQ or mutant groups (Figure 3E).

### 2.4 Overexpression of RHOA counteracts the protumor effects induced by GNAQ inhibition

**Figure 4.**
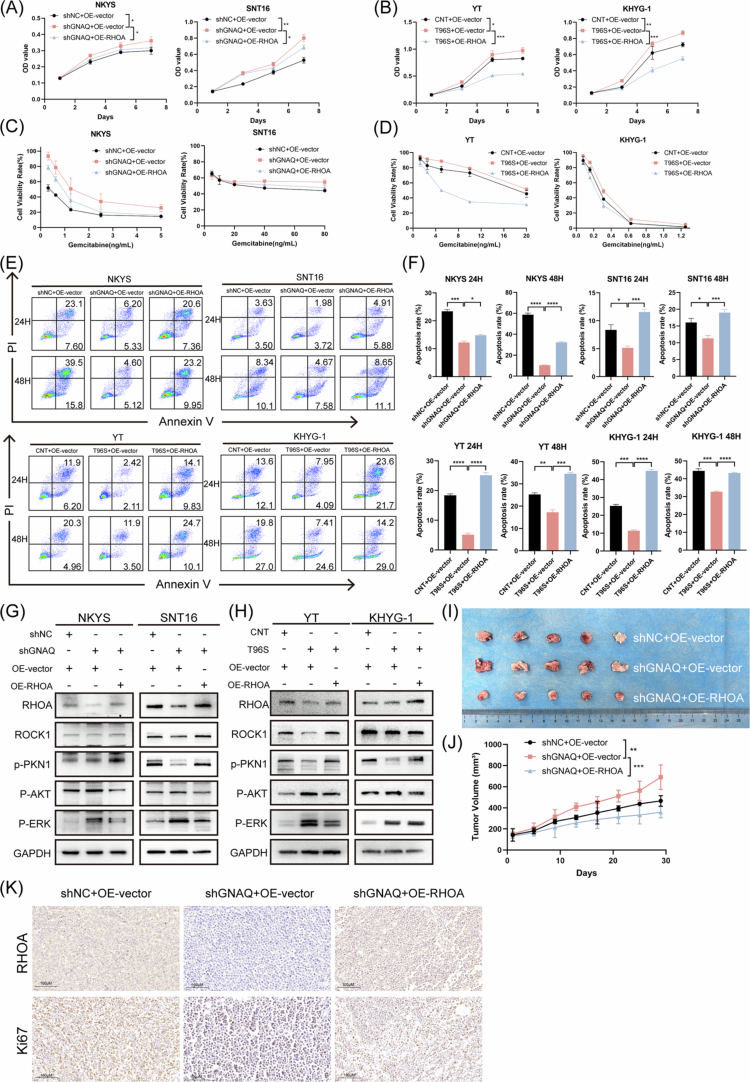
Overexpression of RhoA counteracts the protumor effects induced by GNAQ inhibition. A. Cell proliferation was detected by CCK-8 in NKYS and SNT16. B. Cell proliferation was detected by CCK-8 in YT and KHYG-1. C. Gemcitabine sensitivity was detected by CCK-8 in NKYS and SNT16. D. Gemcitabine sensitivity was detected by CCK-8 in YT and KHYG-1. E. Starvation–induced apoptosis was detected by flow cytometry. F. Starvation–induced apoptosis was analysed. G. Relative protein expression of RHOA pathway–related proteins was detected by western blotting in NKYS and SNT16. H. Relative protein expression of RHOA pathway–related proteins was detected by western blotting in YT and KHYG-1. I. Representative images of the xenograft tumours of NKYS cells. J. Tumour volumes of NKYS cells were analysed. K. Representative IHC staining (400X) of RHOA and Ki67 of the NKTCL xenograft tumour tissues. NKTCL, natural killer/T cell lymphoma; OE–RHOA, over–expression RHOA; CCK-8, Cell Counting Kit-8; no significance; *P＜0.05; **P＜0.01; ***P＜0.001; ****P＜0.0001.

To clarify the cellular function of RHOA in NKTCL, we constructed cell lines that overexpress RHOA. Cell proliferation, apoptosis resistance, and gemcitabine sensitivity were assessed following RHOA overexpression in vitro. As expected, restoring RHOA expression rescued the shGNAQ–induced increase in NKTCL cell proliferation (Figure 4A, B). Similarly, NKTCL cells stably overexpressing RHOA exhibited increased sensitivity to gemcitabine (Figure 4C, D) and increased starvation–induced apoptosis (Figure 4E, F). Furthermore, the expression of RHOA pathway–related proteins was confirmed by Western blotting (Figure 4G, H).

In addition, we assessed the role of RHOA in tumour growth in vivo by constructing NKTCL xenograft mouse models. Nude mice orthotopically injected with shGNAQ YT cells presented greater tumour burdens than control mice did, while RHOA overexpression abrogated the tumour growth–promoting effects of shGNAQ (Figure 4I, J). Furthermore, the expression of Ki67 in YT^sh–GNAQ^ mouse tissues was greater than that in YT^OE–vector^ mouse tissues, and Ki67 expression was lower in the tumour tissues of mice overexpressing RHOA (Figure 4K), indicating that RHOA overexpression inhibited tumour cell proliferation. In summary, these results reveal that activation of the RHOA pathway affects the proliferation and progression of NKTCL cells modulated by GNAQ.

### 2.5 Inhibition of the RHOA pathway induces aggressive biological functions in NKTCL cells

**Figure 5.**
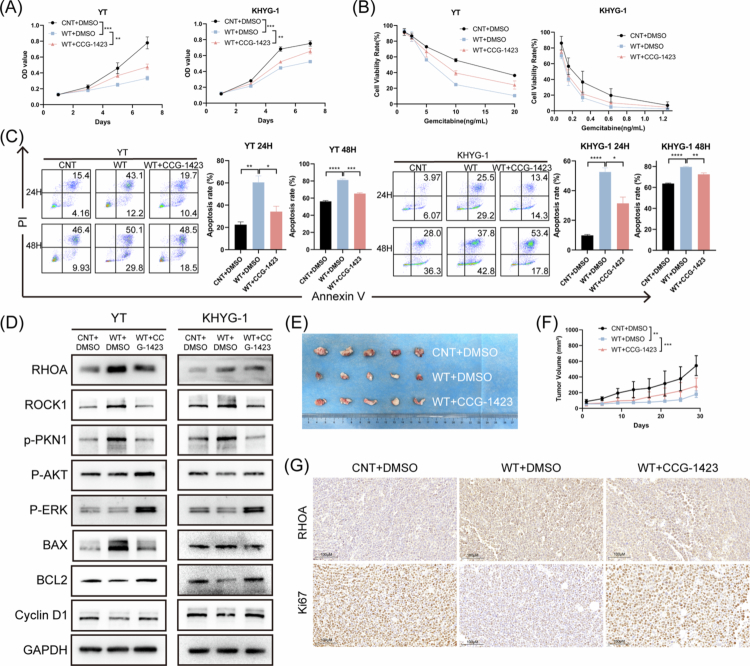
Inhibition of the RhoA pathway induces aggressive biological functions in NKTCL cells. A. Cell proliferation was detected by CCK-8 with inhibition of RHOA pathway. B. Gemcitabine sensitivity was detected by CCK-8 with inhibition of RHOA pathway. C. Starvation–induced apoptosis was detected by flow cytometry with inhibition of RHOA pathway. D. Relative protein expression of RHOA–related proteins was detected by western blotting. E. Representative images of the xenograft tumours of YT cells. F. Tumour volumes of YT cells were analysed. G. Representative IHC staining (40X) of RHOA and Ki67 of the NKTCL xenograft tumour tissues. NKTCL, natural killer/T cell lymphoma; CCK-8, Cell Counting Kit-8; no significance; *P＜0.05; **P＜0.01; ***P＜0.001; ****P＜0.0001.

The effect of RHOA pathway inhibitors on the tumorigenesis and progression of NKTCL cells remains to be explored. Therefore, we assessed cell proliferation, resistance to apoptosis, and sensitivity to gemcitabine in vitro. As expected, inhibition of the RHOA pathway significantly increased the proliferation of NKTCL cells (Figure 5A). Notably, the RHOA pathway inhibitor CCG-1423 mitigated the reduction in NKTCL cell proliferation induced by WT–GNAQ. Consistent with these findings, CCG-1423 also increased the sensitivity of NKTCL cells to gemcitabine (Figure 5B). Furthermore, in experiments assessing apoptosis resistance, CCG-1423 reduced starvation–induced cell apoptosis (Figure 5C). Therefore, we further investigated the expression of RHOA pathway proteins in vitro (Figure 5D).

The role of the RHOA pathway inhibitor in NKTCL tumorigenesis was evaluated in an in vivo NKTCL xenograft model in nude mice (Figure 5E). We found that mice injected with YT^OE–GNAQ^ cells presented a lower tumour burden than those injected with YT^OE–vector^ cells. Treatment with CCG-1423 abrogated the inhibitory effect of WT–GNAQ on tumour growth (Figure 5F). Additionally, Ki67 expression in YT^OE–GNAQ^ mouse tissues was lower than that in YT^OE–vector^ mouse tissues but higher than that in tissues from mice treated with CCG-1423 (Figure 5G). These data highlight the important role of RHOA in the pathogenesis of NKTCL in vivo.

## 3 Discussion

NKTCL is a rare and malignant neoplasm characterised by a highly aggressive clinical course. Despite significant advances in understanding the molecular mechanisms underlying NKTCL in recent years, its rarity and heterogeneity limit the ability to standardise treatment.^17^^,^^18^ The principal strategy of cancer targeted therapy is to selectively attack tumour cells by targeting gene products that play key roles in tumour cell proliferation and survival.^19^^,^^20^ However, this approach remains a significant challenge for gene–based therapies because of tumour heterogeneity and complexity. By integrating the results of our previous report with those of the current study, we identified the phenotypic effects of the GNAQ and T96S mutations on NKTCL.^16^ GNAQ plays a crucial role in the tumorigenesis and progression of NKTCL because of its ability to activate multiple cellular signalling pathways, including those involved in cell proliferation, survival, angiogenesis, and invasion. In this study, we demonstrated the role of GNAQ and the T96S mutation in promoting the development of NKTCL and preliminarily explored the underlying molecular mechanisms involved.

The recent advent of next–generation sequencing techniques has dramatically increased our understanding of the relationships between genomic alterations and various cancer types.^21-23^ Increasing evidence has demonstrated that GNAQ is closely involved in tumour development and progression through multiple molecular mechanisms. Previous work has indicated that the MAPK pathway is a key mediator of GNAQ–mediated oncogenesis.^24^ Mutant GNAQ promotes uveal melanoma tumorigenesis by activating YAP, independent of PLC beta.^25^^,^^26^ These findings suggest that GNAQ plays diverse roles in tumour development and progression through distinct mechanisms. Our previous results revealed that GNAQ deficiency and recurrent somatic GNAQ T96S mutations contribute to the pathogenesis of NKTCL.^16^ GNAQ suppresses NKTCL tumour growth by inhibiting the AKT and MAPK signalling pathways. Moreover, the GNAQ T96S mutation acts in a dominant–negative manner to promote tumour growth in NKTCL. Clinically, patients with GNAQ T96S mutations exhibit poorer survival outcomes. In the present study, we evaluated GNAQ expression in four NKTCL cell lines and selected several for the establishment of stable OE–GNAQ and shGNAQ lines. Additionally, we generated a GNAQ heterozygous model with CRISPR–Cas9-based knock–in technology. Our in vitro results revealed that the overexpression of wild–type GNAQ significantly inhibited the survival of NKTCL cell lines, whereas shGNAQ and the heterozygous GNAQ T96S mutation promoted NKTCL cell proliferation. Furthermore, we demonstrated that shGNAQ and the GNAQ T96S mutation protected NKTCL cells from gemcitabine–induced growth inhibition and apoptosis. In contrast, GNAQ overexpression increased starvation–induced apoptosis and gemcitabine sensitivity in NKTCL cells.

To better understand the role of GNAQ in NKTCL, further studies are necessary to investigate the underlying molecular mechanisms in greater detail. During our investigation of the function of GNAQ in promoting NKTCL, we unexpectedly identified the tumour–suppressor gene RHOA as a potential player. Integrating our findings with those of previous reports, it appears that GNAQ may have significant implications for the regulation of RHOA in NKTCL cells. RHOA is a small G protein that regulates various cellular processes by cycling between an inactive GDP–bound state and an active GTP–bound state.^27^ It is essential for thymocyte development and plays a critical role in the adhesion, polarisation, and migration of T cells.^28^ A prior study demonstrated that inhibition of RHOA function in the thymus resulted in aggressive thymic lymphoma of T–cell origin, suggesting that RHOA–mediated signalling is crucial for T–cell transformation.^29^ Gene set enrichment analysis (GSEA) of our transcriptome sequencing results revealed that the RHOA signalling pathway may contribute to the tumour–suppressive function of GNAQ. We assessed RHOA expression in clinical tissue samples from 56 NKTCL patients and analysed its correlation with clinical characteristics and prognostic indices, including OS and PFS. These findings suggest that RHOA expression could serve as a potential biomarker for risk stratification and prognosis in NKTCL patients.

To further explore the correlation between GNAQ and RHOA and their roles in regulating oncogenic pathways in NKTCL, we conducted functional enrichment analysis of protein‒protein interaction networks with the STRING database, which identified ARHGEF25 as a key protein. On the basis of our experimental data, we propose a working model in which GNAQ upregulates RHOA activation (in its GTP–bound active form) in NKTCL cells. Guanine nucleotide exchange factors (GEFs) catalyse the exchange of GDP for GTP, thereby activating Rho GTPases.^30^^,^^31^ A previous study revealed that RHOA activation occurs indirectly through GNAQ activation, which is mediated by the interaction of the GNAQ subunit with the regulator of G protein signalling (RGS) domain of Rho guanine nucleotide exchange factors.^32^ However, the precise molecular mechanisms by which GNAQ regulates ARHGEF25 in NKTCL remain to be elucidated. The interaction between GNAQ and ARHGEF25 has been extensively studied in vitro with protein extracts from cells through immunoprecipitation, which revealed a direct interaction between these two proteins. Specific GEFs locally activate Rho GTPases (including RHOA, Rac1, and Cdc42) by driving them into the active, GTP–bound state.^33^^,^^34^ Further investigation revealed that GNAQ did not significantly alter the transcription or protein levels of RHOA but rather promoted the binding of GTP to RHOA. Thus, ARHGEF25 mediated the activation of RHOA by GNAQ. Moreover, we transfected NKTCL cells with a luciferase reporter containing SRF–binding elements and performed luciferase assays to evaluate SRF–mediated transactivation. Interestingly, luciferase activity was significantly increased in wild–type GNAQ–expressing cells with increased levels of RhoGEF, whereas no significant effect was observed in the shGNAQ or mutant groups. These findings suggest that ARHGEF25 acts as a signalling hub, mediating the regulation between GNAQ and RHOA in NKTCL cells.

Several studies have shown that RHOA activity regulates cytoskeletal contraction by modulating myosin ATPase activity, with both microtubules and microfilaments playing key roles in these processes.^35^ The activated RHOA pathway is pivotal for tumour cell proliferation, cytoskeletal reorganisation, angiogenesis, and metastasis.^36^^,^^37^ Mutations in GNA13 and RHOA have been reported in Burkitt's lymphoma and diffuse large B–cell lymphoma, in which they promote B–cell lymphoma development.^38^ Additionally, somatic G17V RHOA mutations have been identified in angioimmunoblastic T–cell lymphoma, in which the G17V mutation hyperactivates downstream T–cell receptor signalling through specific binding to the VAV1 protein. Despite these findings, the role of the RHOA signalling pathway in the biological functions of NKTCL cells remains poorly understood.^39^ To investigate whether GNAQ influences the NKTCL phenotype via the RHOA pathway, we generated OE–RHOA cells from shGNAQ and T96S mutant cells and treated WT GNAQ cells with an RHOA inhibitor to observe potential phenotypic changes. Our results demonstrated that OE–RHOA cells exhibited a statistically significant suppression of cellular functions in both the shGNAQ and T96S groups. In contrast, inhibition of the RHOA pathway notably promoted tumour growth.

While this study focuses on the cell–intrinsic functions of the GNAQ/RHOA axis in NKTCL cells, the impact of RHOA signalling within the tumour microenvironment (TME) remains to be elucidated. As a key regulator of cytoskeletal dynamics, RHOA inhibition could affect both immune cells–where it modulates T–cell activation and migration–and cancer–associated fibroblasts (CAFs), which rely on RHOA for matrix remodelling and tumour invasion. Therefore, the overall antitumor effect of RHOA pathway inhibitors likely results from combined direct effects on cancer cells and indirect modulation of the TME. Future studies using co–culture models, immune–competent animals, and spatial omics approaches will be essential to dissect these complex interactions in NKTCL.

## 4 Conclusions

**Figure 6.**
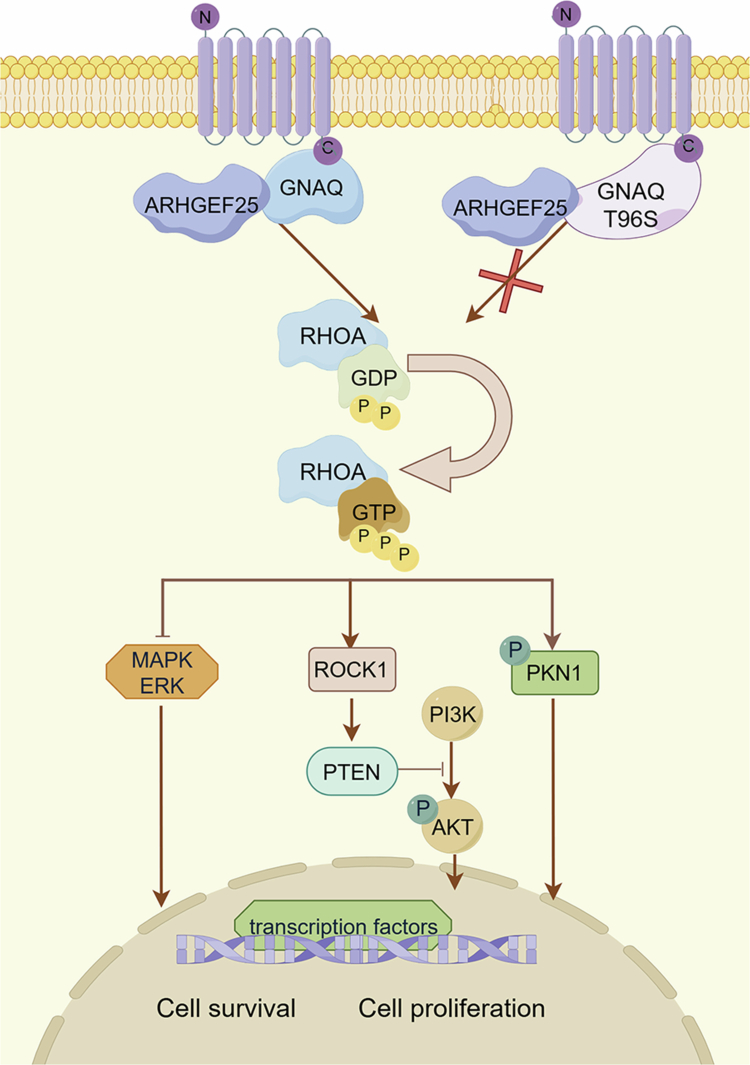
Graphic model of T96S competitive binding with ARHGEF25 to regulate the RHOA pathway in NKTCL cells.

In summary, RHOA is a critical downstream effector of GNAQ in NKTCL. GNAQ promotes RHOA activation through ARHGEF25, which in turn regulates cellular functions by modulating cell proliferation and apoptosis, thereby influencing the progression of NKTCL (Figure 6). These data provide evidence for the involvement of RHOA and GNAQ in tumour progression and suggest that both RHOA and GNAQ may serve as novel prognostic markers and therapeutic targets for NKTCL.

## 5 Materials and methods

### 5.1 Cell lines and culture

NKYS, SNT16, YT and KHYG-1 cells were cultured in RPMI 1640 medium (Gibco, USA) supplemented with 10% foetal bovine serum (Gibco, USA) and 1% penicillin/streptomycin (Invitrogen, California, USA). Additionally, NKYS and KHYG1 cells were maintained in recombinant human interleukin-2 (100 IU/mL, PeproTech, Rocky Hill, NJ, USA). All NKTCL cells were cultured in a 5% CO2 atmosphere at 37 °C.

### 5.2 Construction of stable cell lines

Stable GNAQ–knockdown cell lines were generated with lentiviral constructs expressing short hairpin RNA (shRNA) targeting GNAQ and a negative control. Stable WT GNAQ and T96S GNAQ cell lines were generated with lentiviral constructs of WT GNAQ or T96S GNAQ (WT/T96S) along with corresponding negative controls (CNT). A stable RHOA OE cell line was created with lentiviral constructs of RHOA and the corresponding negative control (OE–vector). Additionally, a stable HA–tagged ARHGEF25 cell line was generated with lentiviral constructs containing ARHGEF25 and the corresponding negative control (OE–vector). All the constructs were designed and provided by Shanghai Genechem Co., Ltd. (Shanghai, China).

### 5.3 CRISPR/Cas9 engineering of GNAQ T96S–mutant tumour cell lines

The NKTCL cell line SNT16 was genetically modified to express GNAQ T96S. Single–cell clones of SNT16 were isolated, and their genotypes were screened by Sanger DNA sequencing and inference of CRISPR edits (ICE) analysis (Synthego) to identify heterozygous GNAQ T96S–targeted clones. Single–cell clones were obtained by limiting dilution, and editing was confirmed by amplifying and sequencing the target loci with specific primers, followed by assessment by Synthego ICE, which evaluates the Indel frequency in Sanger sequencing data. The brief procedures can be found in Additional file 4.

### 5.4 Cell proliferation assay

The cells were seeded in 96-well plates at a density of 1 to 2 × 10^3^ cells per well in 100 μL of cell culture medium or a mixture containing 90 μL of medium and 10 μL of 10 μM CCG-1423 (Selleck, USA). On the indicated days, 10 μL of Cell Counting Kit-8 (CCK-8) reagent (UElandy, China) was added to each well, and the cells were incubated at 37 °C in the dark for 1 hour. The optical density (OD) value was measured with a Multiskan FC microplate reader (Thermo Scientific, USA). The corrected OD value was directly proportional to the number of viable cells. Independent experiments were performed at least three times.

### 5.5 Gemcitabine sensitivity assay

The cells were seeded in 96-well plates at a density of 1 × 104 cells per well in 100 μL of culture medium. The medium contained or did not contain 10 μL of 10 μM CCG-1423, along with 10 μL of gemcitabine (Selleck, USA) at varying concentrations, and was incubated for 48 hours. After incubation, 10 μL of Cell Counting Kit-8 (CCK-8) reagent was added to each well, and the cells were further incubated at 37 °C in the dark for 1 hour. Cell viability was calculated by the following formula: [(OD (gemcitabine treatment)—OD (blank))/(OD (control)—OD (blank))] × 100%. Independent experiments were repeated at least three times.

### 5.6 Cell apoptosis assay

Cell apoptosis was analysed by an APC–Annexin V/PIE Apoptosis Detection Kit (UE Landy, China). After the cells were starved in RPMI 1640 medium for 24 to 48 hours, with or without treatment with 10 μM CCG-1423, the cells from each group were harvested, resuspended in annexin V binding buffer, and stained with annexin V–APC and PIE viability staining solution. The cells were incubated in the dark at room temperature (RT) for 15 minutes. Apoptosis was then assessed with FACSDiva software (version 6.1.2; BD Biosciences, USA) and analysed by FlowJo software (version 10.0; Tree Star, Inc., USA). Independent experiments were repeated at least three times.

### 5.7 Western blotting

Proteins were resolved by SDS‒PAGE with gels of varying concentrations and then transferred onto PVDF membranes (Amersham Biosciences, Piscataway, NJ, USA). The membranes were blocked with TBST buffer containing 5% nonfat milk for 1 hour at room temperature (RT). Next, the membranes were incubated with primary antibodies overnight at 4 °C, followed by incubation with secondary antibodies for 1 hour at RT. A list of the antibodies used can be found in Additional file 2: Table S1. Band images were digitally captured and quantified with a Chemi–Doc™ XRS + system (Bio–Rad Laboratories, Hercules, CA, USA).

### 5.8 mRNA‑seq (mRNA‑sequencing) analysis

Total RNA extraction, mRNA library construction, sequencing, and data analysis were performed by the Novogene Bioinformatics Institute (Beijing, China) according to standard procedures. The brief procedures are listed in Additional file 3. The raw sequencing data are available from the NCBI and are archived under the accession number SRP180943.

### 5.9 Co‑immunoprecipitation (Co‑IP)

After being washed with PBS, the cells were lysed in cold IP lysis buffer (Beyotime, China) supplemented with protease and phosphatase inhibitor cocktail (Beyotime, China) for 30 min on ice. The cell lysates were clarified by centrifugation at 13,000 × g for 30 min at 4 °C. The cell lysate (1000 μg) was combined with the antibody and 50 μL of agarose beads (Beyotime, China) and incubated overnight at 4 °C with rotation. The antigen–antibody–agarose bead mixture was washed 5 to 10 times with cold PBS and eluted prior to SDS‒PAGE. The antibodies used are listed in Additional File 3: Table S3.

### 5.10 Luciferase assay

For the luciferase reporter assay, NKTCL cells were seeded into 12-well plates. The SRF reporter plasmid and the Renilla luciferase plasmid were cotransfected into 293 T cells with Lipofectamine 3000 (Life Technologies). After 12 hours of transfection, the cells were harvested and cultured under detached conditions. The cells were subsequently lysed, and luciferase activity was measured by the Dual–Glo Luciferase Assay System (Promega) with a PerkinElmer EnVision plate reader. Firefly luciferase activity was normalised to Renilla luciferase activity for all samples.

### 5.11 RHOA activity assay

An RHOA Activation Kit (Promega) was used to assess whether RHOA was activated. The GTP–bound form of RHOA was isolated by incubating an equal amount of protein lysate with a predetermined amount of GST–Rhotekin–RBD on a rotator at 4 °C for 1 hour. The beads were then centrifuged, washed, resuspended in 20 μL of loading buffer, and boiled. Western blotting was performed to detect the pulled–down GTP–bound RHOA with an anti–RHOA antibody.

### 5.12 Patients and clinical data

A total of 56 formalin–fixed, paraffin–embedded tumour tissues were collected from NKTCL patients at the First Affiliated Hospital of Zhengzhou University. All the cases were independently reviewed and interpreted by three experienced pathologists, and diagnoses were made on the basis of the latest WHO classification criteria. Immunohistochemical (IHC) staining was performed following standard procedures. The staining intensity and percentage of positively stained areas were evaluated by a pathologist, and the results were verified by two additional pathologists without prior knowledge of the patients' clinical information. Overall survival (OS) was defined as the time from the date of diagnosis to the date of death. Progression–free survival (PFS) was defined as the interval from the date of diagnosis to the date of disease progression or death from any cause. Additionally, the clinical characteristics of the patients and their correlations with RHOA expression are summarised in Table 1. This study was conducted in accordance with the Declaration of Helsinki and approved by the Institutional Research Ethics Committee of the First Affiliated Hospital of Zhengzhou University (approval number: 2024-KY-0090). All patients involved in our study provided written informed consent.

### 5.13 Xenograft tumour assay

All animal experiments were performed in accordance with the guidelines in the Declaration of Helsinki and with approval from the Institutional Review Board of The First Affiliated Hospital of Zhengzhou University (2024-KY-0090). NOD–Scid and BALB/c–Nu nude mice were purchased from GemPharmatech (China). After an 7-day acclimatisation, a total of 1 × 10^7^ NKTCL cells in 400 μL of medium/Matrigel (Corning Incorporated, USA) (1:1 mixture) were injected into the right axillary region by subcutaneous inoculation to establish the following tumour xenograft models: NKYS for NOD–Scid mice and YT for BALB/c‐Nu nude mice. After one week, the mice were randomly assigned to three groups, with a minimum of five animals per group. The mice were given different treatments by intraperitoneal injection (CCG-1423 (0.15 mg/kg) or the corresponding control), with the condition and tumour size monitored every 2 days. The diameter of the subcutaneous graft tumours in the nude mice did not exceed 12 mm. The tumour volume was calculated as follows: volume = ab2/2 (a represents the long diameter, and b represents the short diameter). After 4 weeks, mice were collected and euthanasia was performed for necropsy. All the experimental mice were sacrificed by cervical dislocation and tumours were harvested. The expression of LMP1 and Ki67 was detected by IHC staining.

### 5.14 Statistical analysis

Statistical analyses were performed with SPSS software version 25.0 (IBM Corp., USA) and GraphPad Prism version 8.0 (GraphPad Software, Inc., USA). Independent experiments were repeated at least three times. The data are expressed as the means ± standard deviations of repeated measurements. Comparisons between groups were performed with Student’s t test and analysis of variance. PFS and OS were analysed by the Kaplan–Meier method and log–rank test. The correlations of RHOA expression with clinical features and treatment response were assessed by the χ2 test. A value of *P* < 0.05 was considered statistically significant.

## Experimental model and study participant details

The study was approved by the Institutional Review Board of The First Affiliated Hospital of Zhengzhou University (ethical approval number: 2024-KY-0090). In accordance with the principles outlined in the Declaration of Helsinki, all patients involved in our study provided written informed consent.

## Supplementary Material

Supplementary Material Additional File 4

Supplementary Material Additional File 1

Supplementary Material Additional File 3

Supplementary Material Additional File 2

## Data Availability

The data that support the findings of this study are available on request from the corresponding author.
