# Supplementary material for: GNAQ inhibits tumorigenesis via the ARHGEF25-mediated RHOA pathway in NK/T-cell lymphoma
Source: Cancer Biol Ther. 2025 Dec 9;26(1):2598074. doi: 10.1080/15384047.2025.2598074 (PMC12694899; doi:10.1080/15384047.2025.2598074)
Supplement: Supplementary Material — Additional File 4 [file KCBT_A_2598074_SM1520.docx]

**Methods**

**mRNA-sequencing analysis**

RNA isolation, library construction, and sequencing were performed by the Novogene Bioinformatics Institute (Beijing, China). Total RNAs were extracted using the RNeasy Mini kit (Qiagen) according to the manufacturer’s protocol. The quality and yield of the RNA were assessed by fluorimetry (Qubit, Invitrogen), Nanodrop 1000 spectrophotometry (Thermo Scientific, Wilmington, DE, USA), and gel electrophoresis. RNA integrity was assessed using the RNA Nano 6000 Assay kit with the Bioanalyzer 2100 system (Agilent Technologies, CA, USA). A total of 3 μg of RNA from each sample was used as input material for RNA sample preparation. First, ribosomal RNA was removed using an rRNA Removal kit (Epicentre, WI, USA). Second, sequencing libraries were generated using the Illumina TruSeq RNA Sample Preparation kit (Illumina, CA, USA) according to the manufacturer’s recommendations. To select cDNA fragments between 150 and 200 bp in length, the library fragments were purified with the AMPure XP system (Beckman Coulter, Beverly, USA). Fragments were then amplified by ten cycles of PCR using Phusion DNA polymerase, and libraries were validated with the Bioanalyzer 2100 system (Agilent Technologies, CA, USA). Lastly, the libraries were applied to an Illumina flow cell using the Illumina Cluster Station. After cluster generation, the libraries were sequenced on an Illumina Hiseq 2000 platform, and 100-bp paired-end reads were generated. Bowtie (v2.2.3) was used to build the reference genome index, and TopHat (v2.0.12) was used to align the clean paired-end reads to the reference genome. The read numbers mapped to each gene were counted by HTSeq (v0.6.1), and the expected number of fragments per kilobase of transcript sequence per million base pairs sequenced (FPKM) was calculated. GSEA (version 2.2.4) and gene set collection of KEGG pathways were used for enrichment analysis. The statistical significance of signature enrichment was assessed using 1000-gene-set permutations.
