# Supplementary material for: GNAQ inhibits tumorigenesis via the ARHGEF25-mediated RHOA pathway in NK/T-cell lymphoma
Source: Cancer Biol Ther. 2025 Dec 9;26(1):2598074. doi: 10.1080/15384047.2025.2598074 (PMC12694899; doi:10.1080/15384047.2025.2598074)
Supplement: Supplementary Material — Additional File 1 [file KCBT_A_2598074_SM1519.docx]

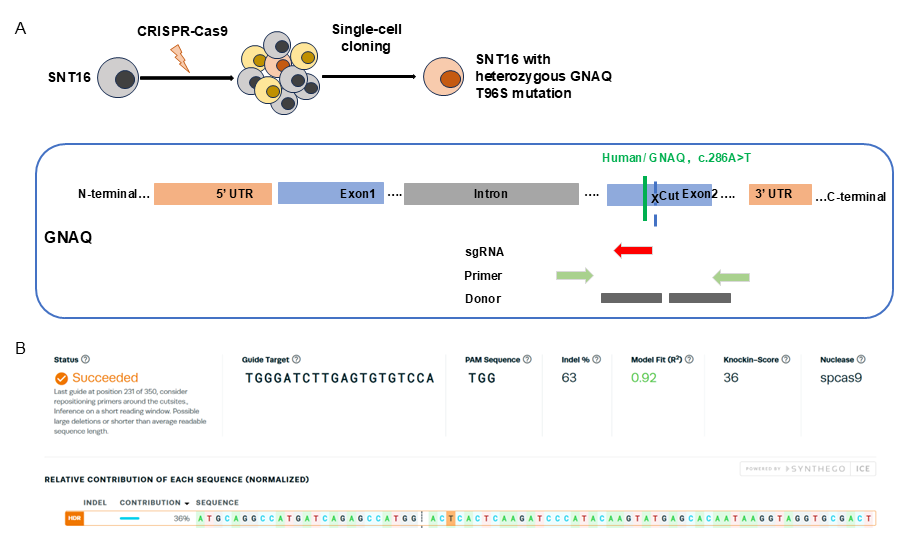


Figure S1. Establishment of SNT16 cell with heterozygous GNAQ T96S mutation.

A. Experimental flow of the approach. A CRISPR–Cas9 complex targeting the specific genomic region was transduced with donor DNA into SNT16 cell using electroporation. Single-cell cloning was performed, and the genomic sequence around the targeted region was determined using capillary sequencing and TA cloning. Clones that carried the targeted heterozygous GNAQ T96S mutations were selected and expanded for subsequent experiments.

B. Single-cell SNT16 clones were isolated, and their genotypes were screened by Sanger DNA sequencing and Inference of CRISPR Edits (ICE) analysis (Synthego) to identify heterozygous GNAQ T96S -targeted clones.


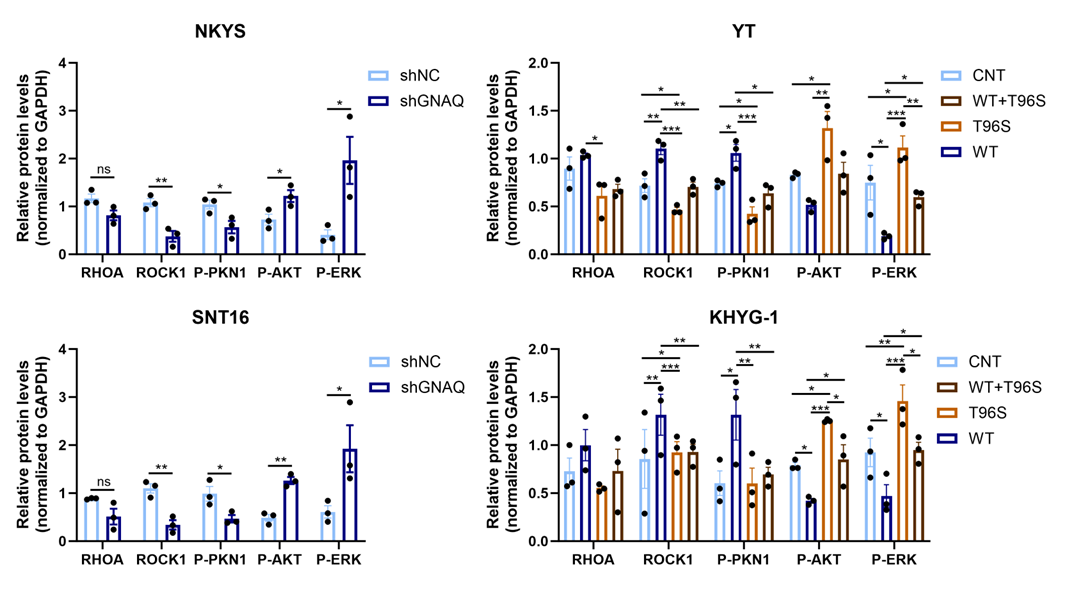


Figure S2. Quantification of Western blot analysis for RHOA downstream effectors.

Protein expression levels were quantified from Western blot images using ImageJ software. Band intensities were normalized to the GAPDH loading control for each sample. Data are presented as the mean ± SD from three independent experiments. Statistical significance was determined by two-tailed paired Student’s t test and one-way ANOVA.


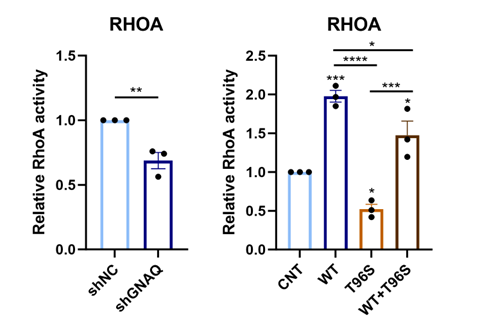


Figure S3. GNAQ regulates RhoA activation in 293T cells.

293T cells were transfected with a control (shNC/CNT), GNAQ-targeting shRNA (shGNAQ), wild-type (WT) GNAQ, or the T96S GNAQ mutant. RhoA activity was measured using a G-LISA activation assay. Data are presented as mean ± SD from three independent experiments. Statistical significance was determined using a two-tailed paired Student’s t-test and one-way ANOVA (*P ≤ 0.05, **P < 0.01, ***P < 0.001, ****P < 0.0001).
