# Supplementary material for: GNAQ inhibits tumorigenesis via the ARHGEF25-mediated RHOA pathway in NK/T-cell lymphoma
Source: Cancer Biol Ther. 2025 Dec 9;26(1):2598074. doi: 10.1080/15384047.2025.2598074 (PMC12694899; doi:10.1080/15384047.2025.2598074)
Supplement: Supplementary Material — Additional File 3 [file KCBT_A_2598074_SM1518.docx]

Table S3. Information of antibodies applied in immunohistochemistry (IHC), co-immunoprecipitation (Co-IP) and western blotting (WB).

| Name (anti-) | Cat No. | Corporation |
| --- | --- | --- |
| GNAQ, 1:1000 (WB), 5µg per 1mg protein (Co-IP) | ab75825 | abcam |
| GAPDH, 1:1000 (WB) | #10494-1-AP | Proteintech |
| RHOA, 1:1000 (WB) | ab54835 | abcam |
| ROCK1, 1:1000 (WB) | #4035 | CST |
| P-PKN1, 1:2000 (WB) | 29107-1-AP | Proteintech |
| P-ERK, 1:2000 (WB) | #4370 | CST |
| P-AKT, 1:2000 (WB) | #4060 | CST |
| HA tag, 1:5000 (WB), 4µg per 1mg protein (Co-IP) | 51064-2-AP | Proteintech |
| RHOA, 1:2000 (IHC) | GB115177-100 | Servicebio |
| Ki67, 1:2000 (IHC) | 28074-1-AP | Proteintech |
| HRP-conjugated Affinipure  Goat Anti-Rabbit IgG (H+L), 1:5000 (WB) | SA00001-2 | Proteintech |
| HRP-conjugated Affinipure  Goat Anti-Mouse IgG (H+L), 1:5000 (WB) | SA00001-1 | Proteintech |
