# Supplementary material for: GNAQ inhibits tumorigenesis via the ARHGEF25-mediated RHOA pathway in NK/T-cell lymphoma
Source: Cancer Biol Ther. 2025 Dec 9;26(1):2598074. doi: 10.1080/15384047.2025.2598074 (PMC12694899; doi:10.1080/15384047.2025.2598074)
Supplement: Supplementary Material — Additional File 2 [file KCBT_A_2598074_SM1517.docx]

Table S1. Univariable analysis and multivariable analysis on progression-free survival (PFS) in NKTCL patients with RhoA expression of enrolled NKTCL patients.

|  | Univariable analysis | | | | Multivariable analysis | | |
| --- | --- | --- | --- | --- | --- | --- | --- |
|  | *P* | HR | 95% CI | *P* | | HR | 95% CI |
| Male sex | 0.88 | 1.996 | 0.902-4.419 |  | |  |  |
| Age＞60 years | 0.786 | 0.882 | 0.357-2.178 |  | |  |  |
| B symptoms | 0.393 | 1.382 | 0.658-2.907 |  | |  |  |
| Stage III/IV | 0.066 | 2.041 | 0.955-4.364 | **0.044** | | 2.192 | 1.021-4.706 |
| High-risk PINK score | 0.499 | 1.293 | 0.614-2.722 |  | |  |  |
| EBV | 0.625 | 0.831 | 0.395-1.747 |  | |  |  |
| Elevated serum LDH† | 0.686 | 1.167 | 0.552-2.467 |  | |  |  |
| Elevated β2-microglobulin | 0.626 | 0.808 | 0.342-1.907 |  | |  |  |
| Ki67 > 50% | 0.835 | 0.919 | 0.416-2.033 |  | |  |  |
| High RHOA expression | **0.046** | 0.466 | 0.220-0.988 | **0.030** | | 0.432 | 0.202-0.925 |

NKTCL, natural killer/T cell lymphoma; PINK, Prognostic Index for Natural-Killer cell lymphoma; LDH, lactate dehydrogenase; β2-MG, β2-microglobulin. Bold means that there is a significant difference. Bold values statistically significant at p < 0.05. LDH lactic dehydrogenase, β2-MG β2-microglobulin, Hb hemoglobin, IFE immunofixation electrophoresis. †Above the upper normal limit for the laboratory of the First Affiliated Hospital of Zhengzhou University.

Table S2. Univariable analysis and multivariable analysis on progression-free survival (OS) in NKTCL patients with RhoA expression of enrolled NKTCL patients.

|  | Univariable analysis | | | | Multivariable analysis | | |
| --- | --- | --- | --- | --- | --- | --- | --- |
|  | *P* | HR | 95% CI | *P* | | HR | 95% CI |
| Male sex | 0.048 | 2.434 | 1.008-5.876 | 0.222 | | 1.768 | 0.708-4.416 |
| Age＞60 years | 0.895 | 0.929 | 0.312-2.77 |  | |  |  |
| B symptoms | 0.395 | 1.451 | 0.615-3.424 |  | |  |  |
| Stage III/IV | 0.301 | 1.58 | 0.664-3.760 |  | |  |  |
| High-risk PINK score | 0.958 | 1.023 | 0.43-2.434 |  | |  |  |
| EBV | 0.890 | 0.942 | 0.400-2.218 |  | |  |  |
| Elevated serum LDH† | 0.446 | 1.4 | 0.590-3.322 |  | |  |  |
| Elevated β2-microglobulin | 0.520 | 1.35 | 0.541-3.371 |  | |  |  |
| Ki67 > 50% | 0.920 | 1.350 | 0.541-3.371 |  | |  |  |
| High RHOA expression | **0.005** | 0.276 | 0.113-0.676 | **0.015** | | 0.317 | 0.126-0.800 |

NKTCL, natural killer/T cell lymphoma; PINK, Prognostic Index for Natural-Killer cell lymphoma; LDH, lactate dehydrogenase; β2-MG, β2-microglobulin.
